# Trends in mortality associated with opening of a full-capacity public emergency department at the main tertiary-level hospital in Tanzania

**DOI:** 10.1186/s12245-015-0073-4

**Published:** 2015-07-22

**Authors:** Hendry R. Sawe, Juma A. Mfinanga, Victor Mwafongo, Teri A. Reynolds, Michael S. Runyon

**Affiliations:** Emergency Medicine Department, Muhimbili University of Health and Allied Sciences, Dar es Salaam, Tanzania; Emergency Medicine Department, Muhimbili National Hospital, Dar es Salaam, Tanzania; Department of Emergency Medicine and Global Health Sciences, University of California San Francisco, San Francisco, CA USA; Department of Emergency Medicine, Carolinas Medical Center, Charlotte, NC USA

**Keywords:** Emergency medicine, Emergency department, Casualty room, Mortality

## Abstract

**Background:**

Emergency medicine is an emerging specialty in Sub-Saharan Africa, and most hospitals do not have a fully functional emergency department (ED). We describe the mortality rates of the Muhimbili National Hospital (MNH) in Dar es Salaam, Tanzania before and after the opening of a full-capacity ED.

**Methods:**

This retrospective study investigated ED and hospital mortality rates for patients admitted to MNH from January 2008 to January 2012. This period represents 2 years before and 2 years after the opening of the full-capacity ED in January 2010. Trained abstractors analyzed patient care logbooks, attendance registers, nurse report books, and death certificates. The January 2008 to December 2009 data are from the limited-capacity casualty room (precursor of the ED), and for February 2010 to January 2012, they are from the new ED. Data are presented as proportions or differences with 95 % confidence intervals (CIs).

**Results:**

During the 4-year study period, the number of visits increased from 53,660 (January 2008 to December 2009) in the casualty room to 77,164 (February 2010 to January 2012) in the new ED. During this time, the overall hospital mortality rate decreased from 13.6 % (95 % CI 13.3–13.9 %) in the January 2008 to December 2009 period to 8.2 % (95 % CI 8.0–8.3 %) in the February 2010 to January 2012 period. The corresponding casualty room and ED mortality rates were 0.34 % (95 % CI 0.25–0.35 %) and 0.74 % (95 % CI 0.68–0.80 %), respectively. In the casualty room, the most commonly reported cause of death was lower respiratory tract infection and least common was poisoning. In the new ED, the most commonly reported cause of death was congestive cardiac failure and the least common was cancer.

**Conclusions:**

The opening of a full-capacity ED in a tertiary-level hospital in sub-Saharan Africa was associated with a significant decrease in hospital mortality. This is despite a small, but significant, increase in the mortality rate in the ED as compared to that in the casualty room that it replaced.

## Background

Emergency medicine (EM) is an integral part of the health care system that represents the first line of care and entry point to most health care facilities across the developed world [1–3]. Generally, emergency care incorporates two major components: medical decision-making and the skills necessary to prevent needless death or disability. These skills include rapid evaluation, resuscitation, and stabilization of patients who have sustained a life- or limb-threatening illness or injury [4]. The quality of emergency care can be used as an indirect marker of the standard of the health care services provided by the hospital and has been shown to be among the major determinants of overall patient satisfaction with the health care system in developed countries [5–7].

In Tanzania, as in other developing countries, EM is an emerging specialty and most hospitals do not have a fully functional emergency department (ED). Instead, they rely on reception areas, referred to as casualty rooms, which are used as a centralized point of entry to the hospital. The staff are tasked with channeling patients to the various departments and wards based on their presenting complaint or referral diagnosis, with little (if anything) done to establish or confirm the diagnosis and provide resuscitative and stabilizing care [8–12]. Most of these casualty rooms are not equipped or staffed to provide rapid evaluation, resuscitation, and management of acutely ill or injured patients. Currently, formal pre-hospital emergency medical services are non-existent in Tanzania, and there is no standardized training or certification for transport personnel. Thus, care received prior to arrival is most often rendered by a Good Samaritan, police officer, or relative. Government-owned ambulances are understaffed, poorly equipped, and mostly stationed within health facilities and used for inter-facility patient transfers.

In January 2010, Muhimbili National Hospital (MNH), a tertiary care center and the main teaching hospital for the country of Tanzania [13], opened the country’s first full-capacity ED. The department is run by local doctors under the training and supervision of board-certified emergency physicians from the USA, Canada, and South Africa. Its presence has improved the quality of care to the patients who were previously being seen at the casualty room of the reception area.

The objective of this study is to describe the demographic patterns of ED and hospital mortality before and after the inauguration of the full-capacity emergency department. The study also reports the documented causes of death and comments on the impact the new facility has had on the emergency care provided in the acute care center of a tertiary-level hospital in a developing country.

## Methods

### Study setting

The study was conducted at the MNH ED in Dar es Salaam, Tanzania. The first of its kind in Tanzania, the ED was established in 2010 via a public-private partnership between the Ministry of Health and Social Welfare and Abbott Fund Tanzania. MNH is the largest tertiary care center in Tanzania, and the ED is the clinical training site for the only emergency medicine residency program in the country.

The department is staffed by interns (fresh graduates from medical school), registrars (independent medical practitioners who have completed internship), and emergency medicine residents (who all have completed 1–3 years work as registrars before joining a 3-year residency program). All of these doctors work under the clinical supervision of the locally trained emergency physicians with support and consultation from a group of board-certified emergency physicians from the USA, Canada, and South Africa.

### Study design

This is a retrospective review of patients presenting to the MNH casualty room for 2 years from 1 January 2008 to 31 December 2009 (before the opening of a full-capacity ED), and then the same review of records was done for patients that presented to the new ED from 1 February 2010 to 31 December 2011. The study excluded neonatal and obstetric patients, as these patients are not admitted into the hospital through the ED or the old casualty room. Trained abstractors analyzed patient care logbooks, attendance registers, nurse report books, and death certificates. All deaths occurring during the study period were reviewed. When possible, death certificates were evaluated to ascertain the documented causes of death.

### Statistics and data analysis

The study data were transferred from the handwritten data forms into an Excel spreadsheet (Microsoft Corporation, Redmond, WA, USA) and imported and analyzed with Epi info (version 7.1.1.14, CDC, Atlanta, GA, USA).

The analysis excluded the month of January 2010 because the department was opened on 25 January 2010 and it did not meet the full month criteria for either the casualty record or emergency department records. The study also excluded patients who were documented as dead on arrival to the casualty room or ED.

Procedure, frequency, and univariate functions were performed to check for outliers and clean the dataset. We calculated descriptive statistics, including the total number of patients seen each year and the number and causes of death in the casualty room and the ED. The casualty room and ED mortality rates for each year were calculated from the number of deaths over the total number of patients seen, respectively. The overall hospital mortality rate for each year was calculated from the total number of deaths occurring each year in the hospital divided by the number of patients who were admitted to the hospital in that particular year.

### Ethical review

This retrospective analysis adheres to the Declaration of Helsinki and was approved by the Muhimbili University of Health and Allied Sciences (MUHAS) Institutional Review Board.

## Results

### Total patient visits to the casualty room and ED

The total number of patients seen at the casualty room for two consecutive years from January 2008 to December 2009 was 53,660, including 29,765 (55.5 %) males and 23,895 (44.5 %) females with a male to female ratio of 1.2:1.

The total number of patients who were seen at the full-capacity emergency department for a period of 24 consecutive months from February 2010 to January 2012 was 77,164, including 44,758 (58.0 %) males and 32,406 (42.0 %) females with a male to female ratio of 1.4:1. The month of January 2010 was excluded in the analysis, because it was a split month as the ED opened on 25 January and before that all patients were seen at the old casualty room. The month of January 2012 was included in the analysis to allow comparison of two 24-month periods before and after the opening of the new ED (Table 1). The total number of patients seen in the first 24 months after the new ED opened increased by 23,504 compared to that in the last 24 months of the casualty room operation, representing a 43.8 % increase in patient volume.

**Table 1:** Total patient visits to the casualty room and emergency department

	Casualty room	Emergency department^a^
	Jan. 2008 to Dec. 2009	Feb. 2010 to Jan. 2012
Male	29,765 (55.5 %)	44,758 (58.0 %)
Female	23,895 (44.5 %)	32,406 (42.0 %)
Total	53,660	77,164

^a^The month of January 2010 was excluded and the month of January 2012 was included to make an analysis of 24 complete months

### Casualty room and ED mortality rates

The total number of deaths recorded in the casualty room from January 2008 to December 2009 was 185, while 568 deaths were recorded in the ED from February 2010 to January 2012, giving crude mortality rates of 0.34 and 0.74 %, respectively (Table 2). This represents an absolute increase in the ED mortality rate of 0.39 % (95 % confidence interval (CI) 0.31–0.47 %) when compared to that of the casualty room (*p* < 0.0001).

**Table 2:** Casualty room and emergency department mortality rates

	Casualty room	Emergency department^a^
	Jan. 2008 to Dec. 2009	Feb. 2010 to Jan. 2012
Total number of deaths	185	568
Total number of patients	53,660	77,164
Mortality rate (95 % CI)	0.34 % (0.25–0.35 %)	0.74 % (0.68–0.80 %)

^a^The month of January 2010 was excluded and the month of January 2012 was included to make an analysis of 24 complete months

### Age distribution of mortality in the casualty room and ED

The age range for deaths recorded in the casualty room was 2 months to 102 years with the mean being 42.95 ± 3.5 years, while the age range for deaths that occurred in the full-capacity ED was 3 months to 92 years with the mean being 39.45 ± 4.5 years. The majority of deaths occurring in both the casualty room and ED were in patients below the age of 40 years (Table 3).

**Table 3:** Age distribution of mortality in the casualty room and emergency department

Age group (years)	Casualty room	Emergency department
	Jan. 2008 to Dec. 2009	Feb. 2010 to Jan. 2012
	No. of deaths (%)	No. of deaths (%)
<5	17 (9.2)	114 (20.1)
5–14	9 (4.9)	79 (13.9)
15–24	17 (9.2)	75 (13.2)
25–34	37 (20.0)	83 (14.6)
35–44	29 (15.7)	60 (10.6)
45–54	17 (9.1)	42 (7.4)
55–64	26 (14.1)	54 (9.5)
65–74	14 (7.6)	26 (4.6)
75+	19 (10.3)	35 (6.2)
Total	185	568

### Reported cause of death in the casualty room and ED

In the casualty room, the most common reported cause of death from January 2008 to December 2009 was lower respiratory tract infections (LRTI) and the least common was poisoning. In the ED, the most common cause of death from February 2010 to January 2012 was congestive cardiac failure and the least common was cancer (Table 4).

**Table 4:** Reported cause of death in the casualty room and emergency department

Casualty room		Emergency department	
Jan. 2008 to Dec. 2009		Feb. 2010 to Jan. 2012	
Cause of death	Number of death (%)	Cause of death	Number of death (%)
LRTI	26 (14.1)	CCF	116 (20.4)
CCF	21 (11.4)	LRTI	71 (12.5)
HIV	19 (10.3)	HIV	56 (9.9)
Malaria	17 (9.2)	RTA	51 (9.0)
Septicemia	16 (8.7)	Trauma (non-RTA)	47 (8.3)
Diarrhea disease	14 (7.6)	Malaria	38 (6.7)
Diabetic coma	13 (7.0)	CVA	32 (5.6)
CVA	12 (6.5)	Upper GI bleeding	30 (5.3)
Neoplasm	11 (6.0)	Diabetic coma	29 (5.1)
TB	10 (5.4)	TB	23 (4.0)
Renal failure	9 (4.9)	Renal failure	20 (3.5)
Upper GI bleeding	7 (3.8)	Septicemia	17 (3.0)
Trauma (non-RTA)	6 (3.2)	Meningitis	15 (2.6)
RTA	2 (1.1)	Hypotension	14 (2.5)
Poisoning	2 (1.1)	Neoplasm	9 (1.6)
Total	185	Total	568

*LRTI* lower respiratory tract infections, *CCF* congestive cardiac failure, *HIV* human immunodeficiency virus, *CVA* cerebral vascular accidents, *TB* tuberculosis, *GI* gastrointestinal, *RTA* road traffic accidents

### Overall mortality rate

The total number of patients who presented to the hospital via the casualty room from January 2008 to December 2009 was 53,660, and the total number of deaths recorded in this population was 7,282 giving an overall mortality rate (casualty plus inpatient) of 13.6 % (95 % CI 13.3–13.9 %).

The total number of patients who presented to the hospital via the ED from February 2010 to January 2012 was 77,164, and the total number of deaths recorded in this population was 6292 giving an overall mortality rate (ED plus inpatient) of 8.2 % (95 % CI 8.0–8.3 %) (Table 5). This represents an absolute decrease in the overall mortality rates of 5.4 % (95 % CI 5.1–5.8 %) between the two time periods (*p* < 0.0001).

**Table 5:** Hospital mortality rate

Period	Jan. 2008 to Dec. 2009	Feb. 2010 to Jan. 2012
Total hospital deaths	7282	6292
Total patients	53,660	77,164
Inpatient mortality rate (95 % CI)	13.6 % (13.3–13.9 %)	8.2 % (8.0–8.3 %)

## Discussion

Our study found a significant decrease in the overall hospital mortality rate for the patients who were admitted during the first 24 months after the opening of the new full-capacity ED as compared with those who were admitted in the last 24 months of the casualty room. The opening of the ED was a major change in the hospital infrastructure and operational processes during the study period and is one of the possible explanations for the decline in hospital mortality. Prior studies have shown that the opening of new EDs with improved infrastructure and operational systems can have positive impacts in care delivery and overall outcome of patients admitted to the hospital [14, 15].

During the study period, there was a 2.1-fold increase in the crude mortality rate for patients seen in the ED as compared with those seen in the casualty room. There are several possible explanations for this finding. First, patients are spending more time in the new ED as they receive more extensive resuscitative care and stabilization as opposed to simply being immediately redirected to other hospital departments or wards. Thus, some of the sickest patients are now dying in the ED when in the past they would have died in the wards or in transit to the wards. Another potential explanation is a change in the referral patterns, with nearby hospitals now sending more of their sickest patients hoping for better care and resources not previously available in the casualty room. Unfortunately, baseline measures of the severity of illness and injury among patients presenting to the casualty room are not available. Despite this increase in mortality rate documented after opening the new emergency department at Muhimbili, overall mortality is still relatively low compared to other emergency departments across Africa [16–19].

Most deaths in the new ED involved patients under the age of 5 years. In contrast, most deaths in the casualty room were patients aged 25–34 years. We believe this shift in age range of the majority of deaths may be due to the fact that during the casualty era, the pediatric patients were prioritized to be rushed to the pediatric wards to be evaluated and treated there. By contrast, these same patients now spend much more time in the new emergency department undergoing rapid evaluation and resuscitative care prior to admission.

Our results include a substantial increase in death due to road traffic accidents (RTA) and trauma within the ED. Similar to the previously discussed patterns seen in the new ED, this change may be due to the fact that currently all trauma patients are being stabilized within the ED as opposed to the casualty room era when such patients were sent directly to the nearby Muhimbili Orthopedic Institute or admitted directly to the surgical unit and reviewed there. Furthermore, prior studies have shown that the incidence of trauma-related deaths is increasing, especially in developing countries, as a result of rapid growth of motorized transport and expansion of industrial production without adequate safety precautions [18, 20–23]. Despite the substantial increase in death due to RTA within the new ED, our data showed a decrease in the mortality rate from other clinical etiologies. We believe that the overall improvement in care, enhanced resuscitative capability (including availability of advanced airway management), ease of obtaining blood and blood products, accessibility to life-saving medications, and efforts of highly skilled clinicians may all be factors contributing to the positive change observed.

Given the multiple possible mitigating factors impacting ED mortality and the many potential benefits to patient care associated with the emergence of the specialty of emergency medicine, we caution against using crude ED mortality rates as a measure of the impact and value of EDs in the developing world. Future work is needed to more rigorously examine the causal factors underlying the associations we observed.

### Limitations

The limitations of this study include the retrospective design and the relative paucity of clinical data available in the attendance registers, nurse report books, and death certificates. Interpretation of the causes of death data is limited by the fact that confirmatory clinical tests are not always available; this was especially true in the casualty room, and autopsies are rarely, if ever, performed. Most importantly, we acknowledge that the study design was limited to characterizing associations and cannot be used to determine causation.

## Conclusions

The opening of a full-capacity ED in a tertiary-level hospital in Tanzania was associated with significant decrease in overall hospital mortality rate. This is despite a small, but significant, increase in the mortality rate in the ED as compared to that in the casualty room that it replaced. Further studies are needed to investigate causal factors of these associations and the predictors of morbidity and mortality among patients presenting to African EDs.

## Abbreviations

CDC: Center for Disease Control
ED: emergency department
MNH: Muhimbili National Hospital
MUHAS: Muhimbili University of Health and Allied Sciences
